# 3D visualization of human colon tissue using a modified CUBIC-based tissue-clearing technique

**DOI:** 10.14440/jbm.2025.0101

**Published:** 2025-02-04

**Authors:** Pavel Pavlov, Andreas Kontny, Neele Wagner, Nikola Kolev, Alexander Zlatarov, Turgay Kalinov, Anton B. Tonchev

**Affiliations:** 1 Department of Anatomy and Cell Biology, Faculty of Medicine, Medical University Varna, Varna 9002, Bulgaria; 2 Department of General and Operative Surgery, Faculty of Medicine, Medical University Varna, Varna 9002, Bulgaria

**Keywords:** Clear unobstructed brain/body imaging cocktails, CUBIC, Tissue clearing, 3D-imaging, Colon, Colorectal cancer

## Abstract

**Background:**

Colorectal cancer represents one of the most common neoplastic diseases worldwide, making it a frequent focus in routine pathological analyses. Visualizing complex three-dimensional (3D) structures, such as nerves within tumors, requires thick tissue sections, which necessitates the use of optical tissue-clearing methods to achieve transparency. However, following tissue clearing, samples typically require advanced imaging techniques such as light-sheet and two-photon confocal microscopy, which are usually unavailable in standard histological laboratories.

**Objective:**

We aimed to demonstrate how a well-established tissue-clearing approach can be adapted for use in a routine histological laboratory, enabling a robust 3D visualization of nerve fibers in samples of both normal human colon and colon cancer tissues.

**Methods:**

We modified the “clear unobstructed brain/body imaging cocktails” method, originally developed for whole-brain imaging in mice, and applied it to human colon tissue samples measuring approximately 10 mm^3^, a standard size typically processed in pathological laboratories.

**Results:**

Our protocol, which integrates a tissue-clearing technique, enabled reliable immunofluorescent visualization of colonic nerve fibers labeled with anti-β_3_-tubulin antibodies. The labeled nerve fibers could be observed using a standard epifluorescence microscope, and high-quality 3D reconstructions were generated through a simple image analysis approach using the open-source software ilastik, which eliminates the need for confocal microscopy.

**Conclusion:**

The proposed steps provide a valuable method for researchers to visualize complex 3D structures, such as neural cells and processes, in both normal and tumor-transformed tissue settings.

## 1. Introduction

Colorectal cancer is one of the most common cancers across the globe. According to GLOBOCAN 2020,^1^ colorectal cancer represents the third most common neoplastic disease in men, following lung cancer and prostate cancer, and the second most common cancer in women, second only to breast cancer. Regionally, data from the National Cancer Registry of Bulgaria^2^ for 2016 and 2017 show a similar pattern, with colorectal cancer ranking the third in frequency among men, second after lung cancer and prostate cancer, and the third among women, after breast cancer and uterine corpus cancer.

Historically, the first mechanistic link between the nervous system and tumor progression was identified as perineural invasion, in which tumor cells spread along nerve fibers.^3^ Recently, reciprocal communication between nerves and tumors has been demonstrated, where the active growth of autonomic nerves into the tumor is related to prognosis in prostate cancer.^4^ Numerous studies in recent years have emphasized the significance of neurogenesis within the tumor microenvironment. A study by Albo *et al*.^5^ on colorectal cancer concluded that neurogenesis is an independent prognostic factor for aggressive tumor behaviors and poorer prognosis.

Routine histochemical techniques applied to paraffin sections up to 5-μm thick offer limited options for examining the nervous system and its components, such as the enteric nervous system (ENS). Examining sections thicker than 50 μm can reveal the structural organization of nerves and enable complete visualization of long processes of ENS cells. The application of three-dimensional (3D) visualization through tissue clearing methods can improve the evaluation of the ENS and its nerve fibers.

“Clear, unobstructed brain/body imaging cocktails,” or CUBIC, was originally developed to visualize the complex 3D anatomy of the brain.^6^ CUBIC is an easy-to-use, low-cost reagent method, in which tissue samples are immersed in amino alcohols to remove lipids, altering the refractive index and increasing transparency.^7^ The CUBIC method can also be combined with indirect immunofluorescent staining, as previously demonstrated for the brain.^8^ Here, we demonstrated the adaptation of this widely recognized tissue-clearing method for routine histological laboratories. In contrast to other methods, such as the ethyl cinnamate (ECi) technique,^9^ which requires dehydration and changes tissue properties (hardening, shrinking), the CUBIC approach preserves the original tissue properties.

Our focus is primarily on the first-generation CUBIC protocol, which differs from the second-generation CUBIC method introduced by Tainaka *et al*.^10^ Other groups have also modified the first-generation CUBIC.^11^ While the second-generation CUBIC has shown promising results in enhancing tissue transparency and improving visualization, our results suggest that the first-generation CUBIC protocol remains a valuable extension of classical histological methods.

Class β_3_-tubulin is specifically localized in neurons, especially in the early phase of neuronal differentiation, and its expression can be used to trace neurons and their axons. In addition, β_3_-tubulin is recognized as a marker for various malignant epithelial tumors and tumors of neuronal origin.^12^ Based on these expression patterns, we selected β_3_-tubulin as the primary marker for our experiment with normal colon tissue adjacent to colorectal carcinoma tissue.

In this study, we aimed to achieve 3D visualization of the intestinal nerves in the human colon. Using transparent, antigen-preserved tissues, we performed immunofluorescence analysis and generated 3D reconstructions of thick sections. After adapting the CUBIC protocol, we presented, to our knowledge, the first visualization of nerve fibers in normal human colonic mucosa following this method.

## 2. Methods

### 2.1. Sample acquisition

Colon tissue was acquired from 10 colon resections performed at St. Marina University Hospital, Varna, Bulgaria (Table 1). The surgeries were performed in 2020, either through open surgery or minimally invasive robotic-assisted laparotomy using the da Vinci Xi system (Intuitive Surgical; USA). Tissue collection was carried out with the approval of the Ethics Committee of the Medical University “Prof. Dr. Paraskev Stoyanov” (protocol #39/07.07.2014). The fresh surgical specimens were promptly delivered to the pathology department for thorough examination. A tissue fragment measuring approximately 1 cm × 1 cm × 1 cm, containing both tumor and normal mucosa, was excised and fixed in paraformaldehyde for 24 – 48 h. After fixation, the tissue samples were washed in phosphate-buffered saline (PBS) 3 times for 30 min each and then stored long-term in a solution containing increasing concentrations of sucrose (15%, 30%) and 0.1% sodium azide.

**Table 1:** Patient data

No	Gender	Tumor Location	Histological type	Grade	LVI	PNI	T	N	M*^a^*	Necrosis	Stromal reaction	Operative procedure
1	F	Flex. coli sinistral	Adenocarcinoma	G2	No	Yes	3	0	x	Yes	Yes	Laparotomia mediana
2	F	Flex. coli dextra	Adenocarcinoma	G2	Yes	No	3	1	x	No	Yes	Laparotomia mediana
3	M	Sigma	Adenocarcinoma	G2	No	No	2	0	x	No	Yes	Laparotomia mediana
4	M	Rectum	Adenocarcinoma	G2	No	No	2	x	x	Yes	Yes	Robot-assisted
5	M	Ascending colon	Adenocarcinoma	G2	No	No	3	x	x	Yes	Yes	Laparotomia mediana
6	M	Rectum	Adenocarcinoma	G2	No	No	3	0	x	Yes	Yes	Laparotomia mediana
7	F	Rectum	Adenocarcinoma	G2	No	No	2	1	x	No	No	Robot-assisted
8	M	Sigma	Adenocarcinoma	G2	No	No	3	x	x	No	Yes	Laparotomia mediana
9	F	Rectum	Adenocarcinoma	G2	No	No	3	1c	x	Yes	Yes	Laparotomia mediana
10	F	Rectum	Adenocarcinoma	G2	No	No	3	0	x	No	Yes	Laparotomia mediana

Notes:

a M refers to distant metastasis, N indicates the regional lymph nodes that are involved, and T represents the size of the primary tumor. Abbreviations: F: Female; LVI: Lymphovascular invasion; M: Male; PNI: Perineural invasion.

### 2.2. Application of a modified CUBIC tissue clearing method

**Figure 1:**
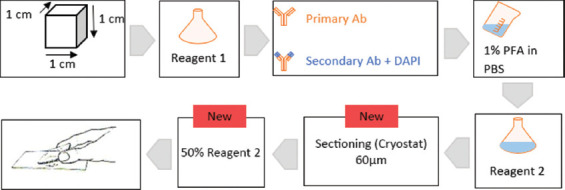
Schematic representation of the main steps for processing human colon tissue samples. Arrows indicate the modified steps implemented in the present study compared to the original CUBIC protocol. Abbreviations: Ab: Antibody; DAPI: 4’,6-diamidino-2-phenylindole; PBS: Phosphate-buffered saline; PFA: Paraformaldehyde.

The CUBIC protocol uses two reagents (reagent 1 and reagent 2), which are applied in series. Each reagent contains a specific set of constituents.^6^ We prepared reagents 1 and 2 according to the original protocol,^6^ without modifications (Table 2). The human colonic tissue samples were submerged in reagent 1, which was refreshed every 2 days for a total of 10 days (Figure 1). Following the incubation in reagent 1 and before the incubation in reagent 2, we applied an immunostaining protocol based on methods described by Nojima *et al*.^8^ and Xu *et al.*,^13^ with modifications to optimize the staining for human colon tissue. After incubation in reagent 1, we washed the tissues 3 times in PBS for 30 min each at room temperature. Next, we blocked non-specific antigens by incubating the tissue in 5% bovine serum albumin (BSA) at room temperature for 2 h. The tissue was then incubated with an anti-β_3_-tubulin primary antibody (rabbit β_3_-tubulin, Covance PRB-435P, BioLegend, USA) at a 1:300 dilution in 5% BSA at room temperature for 96 h. Preliminary experiments comparing incubation times of 24, 48, 72, and 96 h indicated that a 96-h incubation was necessary to achieve optimal staining (Figure 1). Following three washes in PBS (1 h each), we applied the secondary antibody (goat anti-rabbit AlexaFluor 488, Invitrogen A11034; USA) at a dilution of 1:300, along with 4’,6-diamidino-2-phenylindole (DAPI, Invitrogen D1306, USA) at a concentration of 10 μg/ml, in 5% BSA and distilled water for 96 h at room temperature (Figure 1). We then performed a post-fixation protocol using a 1% paraformaldehyde solution, applied three times for 1 h each at room temperature.^8^ After post-fixation, we immersed the tissue in freshly prepared reagent 2[6], diluted 1:1 with distilled water, at 37 °C for 1 h. The samples were then placed in undiluted reagent 2 at 37 °C for an additional 72 h (Figure 1).

**Table 2:** Preparation of CUBIC reagents

Reagent	Substance	Mass fraction (wt%)
1	Urea	25
	Quadrol	25
	Triton X-100	15
	Distilled water	35
2	Urea	25
	Sucrose	50
	Triethanolamine	10
	Distilled water	15

Abbreviation: wt%: Weight percent.

The visualization of cleared tissue blocks, such as those processed in this study, typically requires light-sheet or two-photon confocal microscopy. However, these are not readily available in standard histological laboratories. Therefore, we adapted the protocol for visualization under a standard epifluorescence microscope. To achieve this, we snap-froze (-80°C) the 1-cm^3^ tissue block in Tissue Tek Optimal cutting temperature compound (OCT) and cryo-sectioned it at 100 μm. Sections were thawed in PBS at room temperature for 2 h and transferred to a 1:1 mixture of reagent 2 and distilled water for two 10-min incubations to restore tissue clearing. The sections were mounted on standard microscopic glass slides and cover slipped under a 1:1 mixture of reagent 2 and distilled water (Figure 1).

### 2.3. Image acquisition, processing, and analysis

**Figure 2:**
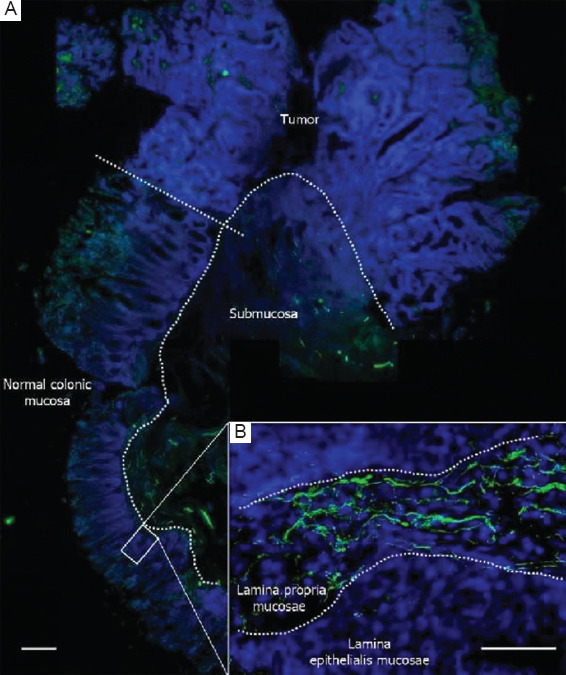
Overview image of the entire specimen. (A) Immunofluorescent visualization of β_3_-tubulin (green) marking nerve structures, and 4’,6-diamidino-2-phenylindole (blue) staining in the intestinal wall, including normal colonic mucosa, submucosa, and the transition to colorectal cancer. Scale bar = 500 μm; Magnification power = 5 × objective. (B) Normal intestinal wall at higher magnification (20 × objective), showing β_3_-tubulin-positive (green) nerve structures in the lamina propria of the normal mucosa. Scale bar = 100 μm.

**Figure 3:**
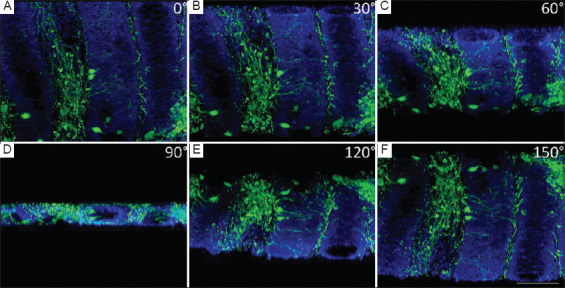
Three-dimensional representation of a portion of the normal intestinal wall, highlighting β_3_-tubulin-positive nerve fibers (green) and nuclei (4’,6-diamidino-2-phenylindole; blue) from different angles. (A) The 0° projection shows the viewpoint along the Z-axis, as seen through the microscope. (B–F) The volume is rotated counterclockwise around the Y-axis in 30° increments (30°, 60°, 90°, 120°, and 150°). The three-dimensional architecture of intestinal crypts and the lamina propria, containin g nerve fibers between individual crypts lined by intestinal epithelium, is visible. Nerve fibers were tracked throughout the reconstructed volume. Scale bar = 100 μm; Magnification power = 20 × objective.

The tissue sections were imaged using an epifluorescence microscope (AxioImager.Z2, Zeiss, Germany) equipped with an X-Cite 120 Metal Halide Light Source. Image acquisition was performed with a Zeiss Axiocam MRm (sensor size: 8.8 × 6.6mm; resolution: 1388 × 1040 pixels). Initially, we captured overview images of the entire specimen at lower magnification to identify the region of interest (Figure 2). Each region of interest was then scanned using an EC Plan-NEOFLUAR 20× objective (Numerical aperture, NA = 0.50) along the Z-axis with a stepwise of 2 μm, generating a z-Stack in.zvi format through the AxioVision v4.8 software (Zeiss, Germany). The raw.zvi image was further processed using the open-source ImageJ/Fiji^14^ and ilastik,^15^ as follows: the two channels (DAPI [blue] and β_3_-tubulin [green]) were separated, and the image data of each channel were loaded into the pixel classification mode of ilastik (v1.3.3). Segmentation of the DAPI (nuclei) and β_3_-tubulin (nerve fibers) was performed, yielding two image stacks of probabilities. These stacks were then imported into Fiji and converted into final binary images using the Otsu algorithm. ilastik feature selection was set to the “recommended” setting with *n* = 7 feature. Using the 3D-viewer plugin “volume mode,”^16^ we created 3D reconstructions of the signals from both channels (Figure 3 and Table 3).

**Table 3:** Workflow for image processing and analysis

Step	Description	Software
Image acquisition	Acquire and save z-stack	Axio Vision

Image processing	Split channels	ImageJ
	Save channels individually as TIF	ImageJ

Pixel classification	New project: Pixel classification (one for each channel)	ilastik
	Choose several features (arbitrary)	ilastik
	Teach segmentation (Label 1, Label 2)	ilastik
	Run feature selection (as recommended)	ilastik
	Choose the feature set with the lowest error	ilastik
	Refine segmentation (in all three axes)	ilastik

Export from ilastik	Export settings: Probabilities	ilastik

Generate binary image	Open segmented images in ImageJ	ImageJ
	Adjust threshold (auto), Otsu algorithm	ImageJ
	Make binary (background NaN)	ImageJ

3D reconstruction	Image – Properties: Enter the pixel size (mm/pixel) and voxel depth (z-step value)	ImageJ
	Open 3D viewer plugin	ImageJ
	Assign color to the first stack	3D viewer
	Add other channels (add – from image)	3D viewer
	Record/manipulate 3D model	3D viewer

Quantitative analysis	Reuse segmented images in ImageJ	ImageJ
	Analyze – Set Measurements: check area fraction	ImageJ
	Analyze – Set Measurements: limit to threshold	ImageJ
	Image – Adjust – Threshold: make sure the segmentation is red; keep the threshold window open	ImageJ
	Analyze – Measure: read the area fraction	ImageJ
	Proceed to the next z-position and measure again	ImageJ

Abbreviations: 3D: Three-dimensional; TIF: Tag Image File.

**Figure A1:**
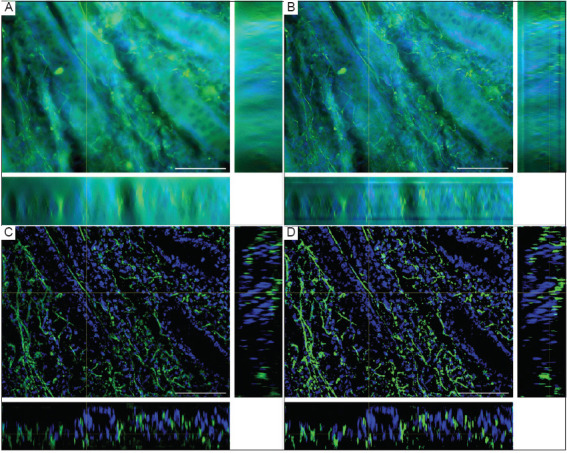
Overview of image data (z-stacks) after CUBIC clearing at different steps of computation leading to the final image. (A) Raw epifluorescence image data. (B) The same image stacked after iterative Richardson–Lucy deconvolution using a theoretical point spread function. (C) Probabilities output after pixel classification using ilastik; (D) the final binary image. All images are shown after min–max stretching for comparison. Scale bar 100 μm; Magnification power = 20 × objective.

An overview of the z-stack (with Orthogonal projections) at three different stages of processing is depicted in Figure A1: (A) raw data, (B) a deconvolved image stack for comparison, (C) segmented images as probabilities, and (D) the final binary image stack.

For quantitative analysis, we measured the nerve fiber density in the reconstructed volume as a percentage of the total volume (volume fraction) by counting segmented pixels and averaging across all slices. This analysis was accomplished by measuring the area fraction of positive versus negative pixels in the binary (segmented) image using the “measure” function in ImageJ (Table 3). The mean value of the area fraction was calculated for each channel per image to determine the volume fraction. Finally, the absolute volume of β_3_-tubulin-positive tissue was calculated by multiplying the total studied volume (volume = length × width × depth) by the volume fraction. For statistical relevance, multiple stacks from several areas were averaged.

## 3. Results

We modified the original CUBIC protocol^6^ to optimize its use for a 3D visualization of human colon tissue without the need for light-sheet confocal microscopy. The optimization included modifications to the following steps: (i) we found that a 10-day incubation in CUBIC reagent 1 (longer than the incubation times reported in^6^,^8^) was necessary to achieve satisfactory transparency, compared to the shorter incubation time of less than a week used for mouse brain tissue;^6^ (ii) longer incubations with primary and secondary antibodies compared to previous CUBIC procedures^6^,^8^ to achieve sufficient antibody penetration into the human colon tissue; and (iii) after incubation with reagent 2, we cryo-sectioned the tissue block to allow for the observation of serial sections under standard epifluorescence. Reagent 2 was used as a mounting medium for the sections to equalize the refractive index. The main differences between our protocol and previous CUBIC studies^6^,^8^ are summarized in Table 4.

**Table 4:** Comparison of the main steps of the CUBIC procedure as applied in previous studies and the modifications (in blue) introduced in the present study

Susaki *et al.* 6	Nojima *et al.* 8	Xu *et al.* 13	Our procedure
Tissue: Mouse brain	Tissue: Human lung and lymph node	Tissue: Mouse brain	Tissue: Human colon
Fixation	Fixation	Fixation	Fixation
Tissue block	Tissue block	Tissue block	Tissue block
Wash	Wash	Wash	Wash
Reagent 1: 3 days	1:1 reagent 1:dH_2_O (1 day)	1:1 reagent 1:dH_2_O (3 h)	1:1 reagent 1:dH_2_O (2 h)
Reagent 1: 3–4 days	Reagent 1: 3–7 days	Reagent 1: 7 days	Reagent 1: 10 days
Wash	Wash	Wash	Wash
Cryo-protection, freeze-thaw cycles	Cryo-protection, freeze-thaw cycles	No freeze-thaw cycles	No freeze-thaw cycles
Incubation of primary antibody: 72 h	Incubation of antibody: 72 h	Incubation of primary antibody: 24 h	Incubation of primary antibody: 96 h
Wash	Wash	Wash	Wash
Incubation of secondary antibody: 72 h	No secondary antibody used	Incubation of secondary antibody: 24 h	Incubation of secondary antibody: 96 h
Wash	Wash	Wash	Wash
No post-fixation	Post-fixation: PFA	No post-fixation	Post-fixation: PFA
Reagent 2: 24–36 h	1:1 reagent 2:dH_2_O (24 h)	1:1 reagent 2:dH_2_O (24 h)	1:1 reagent 2:dH_2_O (1 h)
-	Reagent 2: 24 h	Reagent 2: Every 2^nd^ day	Reagent 2: 72 h
-	-	-	Snap-freeze, section on a cryostat
-	-	-	1:1 reagent 2:dH_2_O, mount
Observation	Observation	Observation	Observation

Abbreviations: dH_2_O: Distilled water; PFA: Paraformaldehyde.

**Figure 4:**
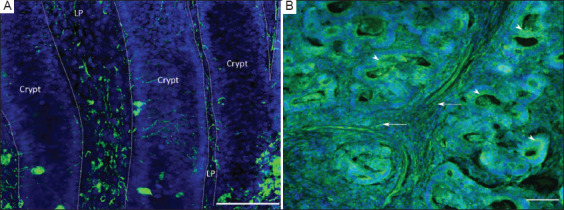
Staining of nerve fibers in the human colon. (A) Immunofluorescent staining of β_3_-tubulin (green) highlighting nerve structures in normal colon mucosa. Scale bar = 100 μm; Magnification power = 20 × objective. (B) Staining in colon cancer tumor tissue. Nuclei are counterstained with 4’,6-diamidino-2-phenylindole (blue). Expression of β_3_-tubulin is observed in carcinoma cells (arrowheads) and axons (arrows). Scale bar = 100 μm; Magnification power = 10 × objective. Abbreviation: LP: Lamina propria.

By applying the optimizations described above, we were able to detect a robust signal for β_3_-tubulin in the lamina propria of the normal gut wall (Figure 4A) as well as in the tumor (Figure 4B). Using only standard epifluorescence, we attained a strong signal at a depth of 300–500 μm from the surface of the tissue block, as well as in deep sections, 3–4 mm from the surface. The fluorescent z-stacks were easily transformed into 3D reconstructions using open-source platforms such as Fiji and ilastik (Figure 3).

The computation was performed on a commercially available PC system using Windows 10, equipped with an Intel Core i9-10900K CPU, Nvidia RTX2080 graphics card, and 32 GB of RAM. The step with the longest computational duration was the segmentation process, which took approximately 15 min per channel (30 min in total) on this system and was performed for each plane in the z-stack. A two-channel, 50-slice z-stack can be processed in approximately 1 h, from acquisition to 3D reconstruction and quantitative analysis. After processing the raw data image in ilastik and ImageJ, we were able to detect the signal throughout the cleared tissue samples, enabling optimal quantitative analysis and 3D reconstruction. In Figure A1, we show the difference between the commonly used Richardson–Lucy deconvolution^17^ and ilastik-based pixel classification. Pixel classification in ilastik results in robust segmentation, facilitating the final 3D reconstruction and quantitative analysis in a simple and efficient process.

The quantitative evaluation showed that 77,492 ± 2,017 μm³ of the studied volume 11,410,268 μm³ ≈ 0.11 mm³ were positive for β_3_-tubulin. This observation represented 0.68% (± 0.02% standard error of the mean) of all pixels in the z-stack labeled for β_3_-tubulin. The chosen volume contained intestinal mucosa (lamina epithelialis and lamina propria) with β_3_-tubulin-positive nerve fibers and/or neuroendocrine cells.

## 4. Discussion

To the best of our knowledge, this is the first study to apply the CUBIC method for optical clearing of human colon tissue. Our aim was to develop an approach that allows for a robust 3D visualization and analysis of nerves in the human colon within a standard laboratory setting. Our protocol consisted of four major steps: (i) application of an optical tissue clearing approach on thick tissue blocks; (ii) optimization of indirect immunofluorescence detection of nerves within the block; (iii) cutting the block into slices that are thin enough for observation under standard epifluorescence microscope but still thick enough to allow for visualization of the entire crypt, including its lamina propria and nerve fibers; and (iv) the use of open-source tools for processing epifluorescence images, enabling rapid 3D reconstruction and analysis.

Initially, we visualized the innervation of the human colon using conventional immunofluorescence staining. We successfully stained nerve structures in both normal colonic tissue and tumor tissue, as well as neuroendocrine cells in normal crypts and tumor cells (Figure 4).

For further investigation and improved 3D reconstruction, we increased the section thickness to more than 50 μm, which led to a decrease in image quality when using standard methods. To address this issue, we modified and applied the CUBIC tissue clearing method, successfully processing tissue slices up to 100-μm thickness.

We demonstrated that β_3_-tubulin staining yielded robust results following CUBIC treatment, and it may serve as a control for future investigations of the human colon wall and tumors when using other antibodies for labeling.

As an optical clearing approach, we chose the CUBIC method^6^,^8^,^10^ because it relies solely on incubations with chemical compounds, without the need for additional expensive equipment such as platinum electrodes, which are not commonly available in standard histological laboratories. We found that the total duration of the protocol (including CUBIC reagents 1 and 2, as well as primary and secondary antibody staining) took approximately 3 weeks. Although this duration is longer than the 1-week processing time for paraffin blocks, it allows for tracking of nerve fibers in the 3D space of the colon mucosa at a depth and level of detail that cannot be achieved with standard paraffin sections.

When comparing our CUBIC approach to other established methodologies, such as the ECi technique,^9^ several differences in outcomes and efficiency emerged. ECi clearing achieves tissue transparency by first dehydrating the tissue sample and then incubating it with ECi, a non-toxic organic compound. This method preserves antigenicity well, enabling clear and detailed imaging, though it results in some tissue shrinkage and hardening. In contrast, tissue processed using our CUBIC method preserved the integrity of the intestinal crypts, confirming that our method does not cause major visible structural damage (Figure 3). At the time of our study, our laboratory had access only to epifluorescence imaging, which limited our ability to validate the 3D images using other methods, such as confocal microscopy or light-sheet fluorescence microscopy (LSFM). We recognize this as a limitation of our results, but we also acknowledge that the lack of access to other imaging modalities was a driving factor behind the conception of this study.

The ability to effectively and rapidly visualize nerve structures in both tumor and normal tissues within the colon is an important methodological advancement that will aid in addressing scientific questions regarding the interactions between innervation and tumor tissue.^3^-^5^ For practical purposes, it is essential that such analyses can be performed without the need for complex and expensive equipment, which is typically only available at specialized research centers. Our objective is to demonstrate that tissues samples up to 100 μm in thickness can be observed using standard laboratory equipment. While other studies employing the CUBIC method work with tissues several millimeters thick, they often rely on specialized imaging techniques such as LSFM or laser confocal microscopy (LSM).^8^ The staining and image analyses workflow reported here, which used only epifluorescence microscopy, would benefit from validation using LSFM and/or LSM. Future studies are necessary to confirm that the limitations of the current protocol (specifically, the small section thickness) do not hinder the reproducibility of 3D image generation, and whether it can yield results comparable to those obtained using LSFM and/or LSM. In addition, the protocol needs to be adapted for other nerve or glial markers to investigate the reciprocal interactions between nerve/glia and tumor cells in the cancer microenvironment.

In our workflow, the primary image processing step after acquisition was pixel classification in ilastik, rather than image deconvolution. In fact, deconvolution alone (Figure A1B) is not sufficient for high-quality 3D reconstruction and it is technically demanding in terms of computational power. Therefore, in our protocol, we omitted the deconvolution step and directly proceeded with pixel classification, which made the processing of acquired image stacks both faster and more robust.

Routine pathological examination includes screening the entire slide to identify expression hotspots of the relevant marker. After locating the area with the most positive structures, adjacent fields are examined to determine the degree of expression. If a hotspot cannot be identified, 10 high-power fields are analyzed. In contrast, when examining the intestinal wall near a tumor mass, a more representative approach involves selecting random fields to gain a general understanding of the changes in composition and the percentage ratio of the nervous elements of the ENS. Using our method, we examined a volume 7 times larger at each field position. This approach allows for a more comprehensive analysis, particularly in the case of neural elements, as it enables the tracing of nerve fibers over longer distances and provides a more details of their 3D architecture.

## 5. Conclusion

This study demonstrated a CUBIC-based tissue clearing protocol for human colorectal cancer tissue, enabling 3D visualization of nerve structures using standard laboratory equipment. We used prolonged incubation periods and antibody staining to achieve immunofluorescent labeling in both normal and tumor tissues. This work provides an accessible and cost-effective framework for visualizing neural structures in colorectal cancer and potentially other pathological conditions. CUBIC-based clearing can be implemented for further investigation of neural-tumor interactions and investigation of other markers and tissues.

## Data Availability

The original data presented in the study are included in the article. Further inquiries can be directed to the corresponding author.
